# Reducing Inappropriate Abdominal X-ray Requests in a Surgical Admissions Unit: A Quality Improvement Project

**DOI:** 10.7759/cureus.93315

**Published:** 2025-09-26

**Authors:** Michael Barrington, Pranu Ragatha, Emily-Jane O'Malley, Rob Bethune

**Affiliations:** 1 General Surgery, Royal Devon University Healthcare NHS Foundation Trust, Exeter, GBR; 2 Colorectal Surgery, Royal Devon University Healthcare NHS Foundation Trust, Exeter, GBR

**Keywords:** abdominal x-rays, axr, irefer guidelines, quality improvement research, surgical acute abdomen

## Abstract

Introduction: Abdominal X-rays (AXRs) are a common investigation for acute abdominal pain. Computed tomography (CT) imaging has largely replaced AXRs as the recommended investigation of acute abdominal pain due to the greater detail and improved diagnostic information it can provide. This project aimed to reduce the number of inappropriate AXR imaging requests in a surgical admission unit (SAU).

Methods: AXR requests were compared against national radiology guidelines (iRefer) to assess if they were inappropriate. Initial baseline data were collected over three months. Three interventions were introduced to improve the rate of inappropriate requests made: (1) developing locally agreed guidelines for AXR requests, (2) placing guideline posters in the surgical assessment unit, and (3) altering the electronic AXR order form. Data were collected after each intervention period. The number of CT scan requests was collected before and after intervention 3 to assess for concurrent change in volume.

Results: At baseline, 69% of AXR requests were considered inappropriate; the majority of requests made were for bowel obstruction (BO, 60%). Overall rates of inappropriate requests improved to 50% after intervention 1, 41% after intervention 2, and 11% after intervention 3. CT scan requests remained similar pre- and post intervention 3.

Conclusion: The project has reduced the inappropriate use of AXRs following the implementation of three interventions.

## Introduction

Acute abdominal pain is a common presentation to surgical units and emergency departments (EDs) that can represent a wide range of pathologies [1]. A variety of different medical imaging modalities can be utilised as part of the workup to formulate a diagnosis and guide management plans. Plain film radiography has been used as a form of medical imaging since its discovery in 1895 by Wilhelm Röntgen, with abdominal X-rays (AXRs) being adopted as an investigation for the acute abdomen presentation [2]. However, multiple studies have shown that AXR lacks both sensitivity and accuracy, with a high false-negative diagnostic rate in the assessment of acute abdominal pain [3]. With increasing availability and accuracy of newer modalities such as computed tomography (CT) imaging, AXR has largely been superseded as the ideal investigation due to the greater detail and diagnostic value provided by CT [4]. Only a few clinical indications remain where AXR is a recommended imaging modality for acute abdominal pain, and the Royal College of Radiologists (RCR) has produced national guidelines to outline these, known as the iRefer guidelines [5].

A recent report by the National Confidentiality Enquiry into Patient Outcome and Death (NCEPOD), examining the current management of bowel obstruction (BO), showed AXRs were only able to aid in diagnosing BO in 32% of patients undergoing this imaging modality [6]. The report found that AXRs did not provide an accurate aid in decision-making or contribute to further management planning, overall recommending that CT scanning should be the primary investigation when suspecting BO. This was similarly seen in the National Audit of Small Bowel Obstruction (NASBO) that noted 65% of patients had both AXR and CT imaging performed, with the similar recommendation of CT being first-line to minimise unnecessary irradiation and delays in diagnosis for patients [7]. The iRefer guidelines include BO within the indications for AXR imaging but cite specific circumstances only, such as sigmoid volvulus or suspected toxic megacolon, where it may be of benefit over definitive testing such as CT [5]. Despite these recommendations, AXRs are still commonly used in EDs and surgical admission units (SAUs) for the assessment of acute abdominal pain.

The aim of the project was to reduce the rate of inappropriate AXR imaging requests in a surgical admissions unit to 10% of total requests within two years.

## Materials and methods

Data collection took place at Royal Devon and Exeter Hospital, Exeter, UK, a large teaching hospital in an SAU receiving referrals for adult patients from general practitioners and the ED. The hospital had recently introduced an all-encompassing electronic patient record system (EPIC, EPIC Systems Corporation, Verona, WI) [8]. This incorporates all aspects of patient care within the system, including doctor, nursing, and auxiliary notes, investigations and imaging requests, drug charts, and vitals. EPIC provides built-in report tools that can identify imaging requests within specific hospital departments, including clinical details provided on the requests. These reports were used to identify all AXR requests on SAU in the respective data collection time periods. Requests were then assessed on the clinical details provided and reviewed against the iRefer guidelines to determine if an inappropriate indication was provided (Table 1). The total volume of AXR requests was concurrently assessed to see if the project had an impact.

**Table 1:** Summary of RCR iRefer guidelines for the use of AXR imaging in adults AXR: abdominal X-ray; RCR: Royal College of Radiologists Source: [4,7]

Summary of iRefer guidelines indications for AXR imaging
Clinical suspicion of bowel obstruction (specific circumstance)
Ingestion of metallic/radiopaque foreign bodies (including sharp or poisonous such as batteries or magnets)
Acute exacerbation of Inflammatory bowel disease
Renal stones follow-up
Constipation (specific circumstance)
Palpable mass (specific circumstance)
Acute and chronic pancreatitis (specific circumstance)
Blunt or stab abdominal injury (specific circumstance)

Baseline data were collected retrospectively to review the current level of inappropriate AXR requests and assess the extent of the problem. This was conducted over a three-month duration between October and December 2020 and included all adult patients reviewed on SAU. The duration of baseline data collection was limited due to the implementation of EPIC at this time point, and therefore, prior data could not be collected using the EPIC report features.

Intervention 1: local guidelines

The surgical consultants agreed that a high rate of inappropriate AXRs was being requested. A locally agreed consensus for AXR indications in patients with acute abdominal pain was summarised and is shown in Table 2.

**Table 2:** Locally agreed guideline indications for AXR requests AXR: abdominal X-ray; IBD: inflammatory bowel disease Table credits: M Barrington

Local guidelines for AXR indications
Renal stone follow-up
Gastrograffin follow-through study
Foreign body
Acute IBD flare
Specific consultant request

Intervention 2: posters

To build on the improvements of the first intervention, we created posters to serve as aide-mémoires of the agreed indications for AXR in Table 2 for the surgical juniors on the SAU. These posters included the new local guidelines and were placed in visible areas of the admissions unit to provide continuous reminders to clerking doctors.

Intervention 3: EPIC request form

**Figure 1:**
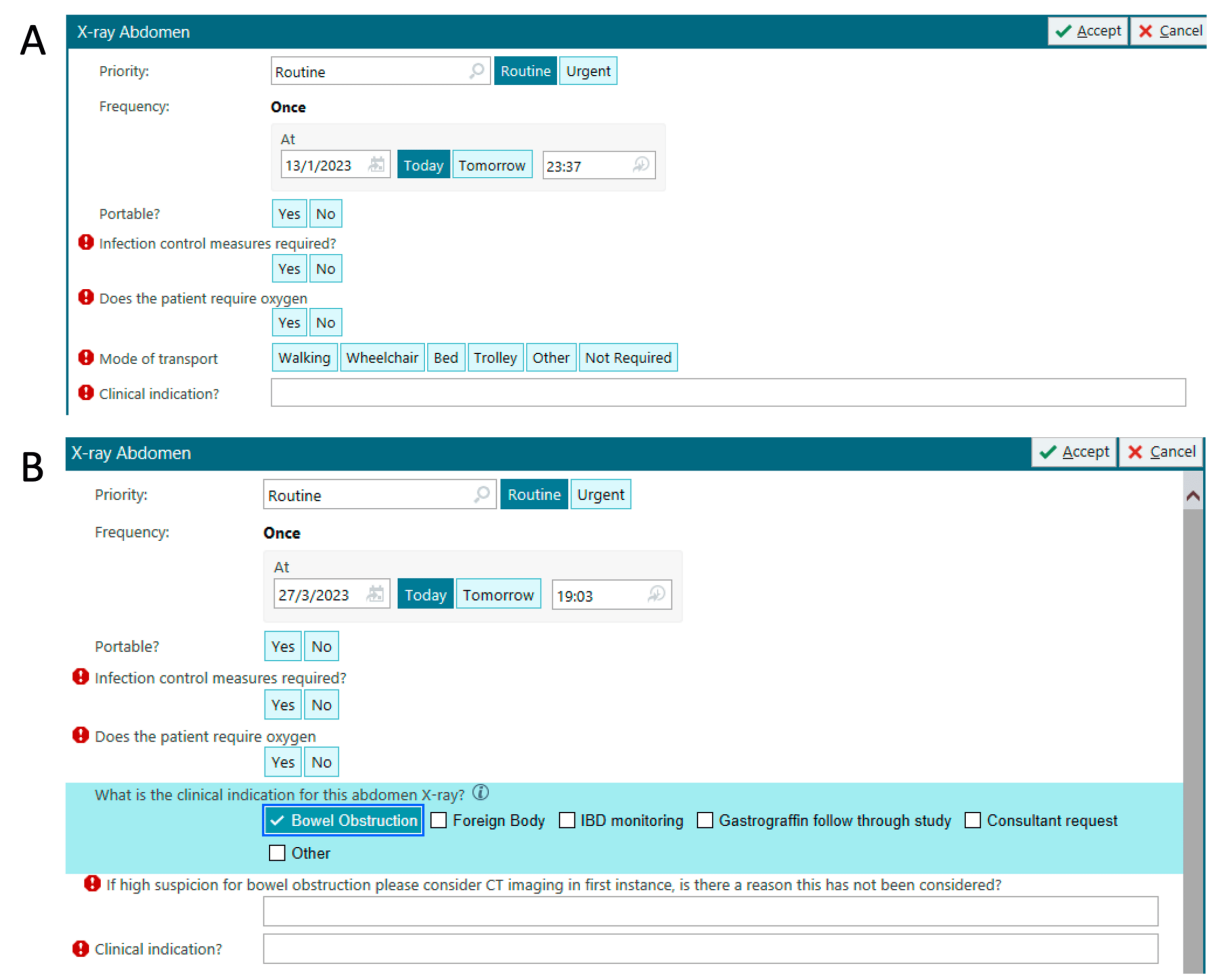
AXR request form on EPIC system demonstrating pre- (A) and post- (B) intervention appearances. AXR: abdominal X-ray; IBD: inflammatory bowel disease

The original EPIC request form for AXR is shown in Figure 1a. Intervention 3 involved altering this electronic form to incorporate the locally agreed guidelines (Figure 1b). This would highlight to the ordering clinician the recommended indications for AXR imaging and to the vetting radiographers to assess its appropriateness.

Following each intervention, data collection was performed retrospectively on a monthly basis using the EPIC reports to assess the impact each intervention had on the rate of inappropriate AXR requests and the volume of AXR requests made against the locally agreed guidelines.

Alteration of the EPIC request form required engagement with our radiology department. The radiologists were concerned that it might lead to an increase in the volume of CT scans as a response to not using AXR in certain cases. As such, CT scan volume was monitored concurrently with intervention 3 to assess if the EPIC form change led to an increase in CT requests within the SAU. Alongside this, though the local guidelines did not include BO within the agreed indications, to allow changes to the order form on EPIC, this was required to remain as an option on the form. On selecting this option, however, it would open a new line in the order form that suggests CT scanning should be considered as the first line for suspected BO.

Statistical analysis was performed using a paired two-tailed Student's T-test in Microsoft Excel (Microsoft Corp., Redmond, WA). Run charts were created using the AQUA SPC chart (Advancing Quality Alliance (AQUA), Sale, England) [9].

## Results

Baseline data identified that 90 AXRs were requested during these three months; correlation with the iRefer guidelines showed that 63 (69%) were inappropriate requests. A breakdown of the individual request clinical details (Table 3) shows that BO was the main indication with 54 requests (60%).

**Table 3:** Breakdown of individual requests for AXR imaging AXR: abdominal X-ray; IBD: inflammatory bowel disease; N: sample size

Indication	Baseline		Intervention 1		Intervention 2		Intervention 3	
	N	%	N	%	N	%	N	%
Bowel obstruction	52	57.7	25	48.1	113	23.7	11	5.56
Renal stones	17	18.9	15	28.8	172	36.1	96	48.5
Gastrograffin	8	8.9	6	11.5	135	28.4	59	29.8
IBD	2	2.2	0	0	3	0.63	1	0.51
Foreign body	0	0	4	7.7	31	6.51	20	10.1
Constipation	4	4.4	2	3.8	11	2.31	9	4.55
Abdominal pain	4	4.4	0	0	7	1.47	1	0.51

**Figure 2:**
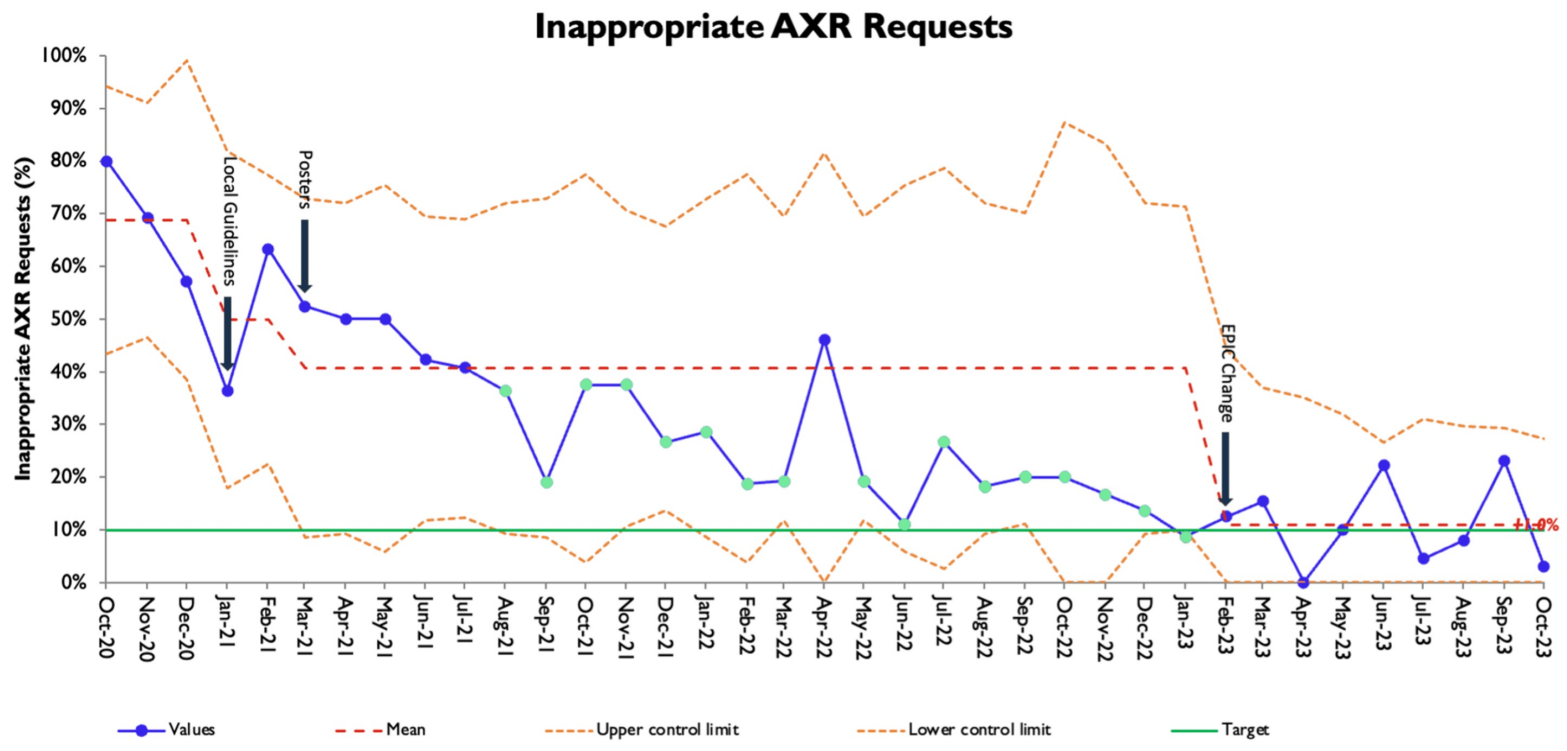
Monthly run chart of inappropriate requests for AXRs; implementation of interventions are indicated by arrows. AXR: abdominal X-rays

Subsequent data collection after each intervention is shown in the monthly run chart with correlation to the locally agreed guidelines for request inappropriateness (Figure 2). Following intervention 1, inappropriate requests reduced to 50% (n=27). This then improved to 41% (n=133) following intervention 2 and finally to 11% (n=22) following intervention 3. Baseline data included 12 data points, as the data was collected on a weekly basis; graphs have been amalgamated on a monthly basis to allow easier interpretation.

A breakdown of the requested indications for each collection period is shown in Table 3. Bowel obstruction was shown to reduce from 54 (60%) requests at baseline to only 11 (5.56%) requests in the third post-intervention data collection. Abdominal pain requests decreased from four (4.4%) to one request (0.51%). The most common requests following intervention 3 are now for gastrograffin follow-through studies (29.8%) and renal stone monitoring (48.5%). Constipation remained similar at the start and end of the project, with nine requests (4.55%). The foreign body request rate has increased over the project duration from no requests at baseline to 20 (10.1%).

The data are presented with the individual number of requests made for that period of data collection (N) and the percentage (%) of those request types from the total data period requests (Table 3).

Volume of AXR requests is shown in Table 4. The number of AXR requested per month for each intervention shows an overall reduction following each successive intervention, with 30 requests per month at baseline and reducing to 22 requests per month after intervention 3. There was an increase in AXR per month following the introduction of intervention 3, increasing from 20.7 to 22 requests.

**Table 4:** Number of AXRs requested per intervention period and volume per month AXR: abdominal X-rays

	Baseline	Intervention 1	Intervention 2	Intervention 3
Number of AXRs requested	90	52	476	198
Time period (months)	3	2	23	9
Number of AXRs/month	30	26	20.7	22

**Figure 3:**
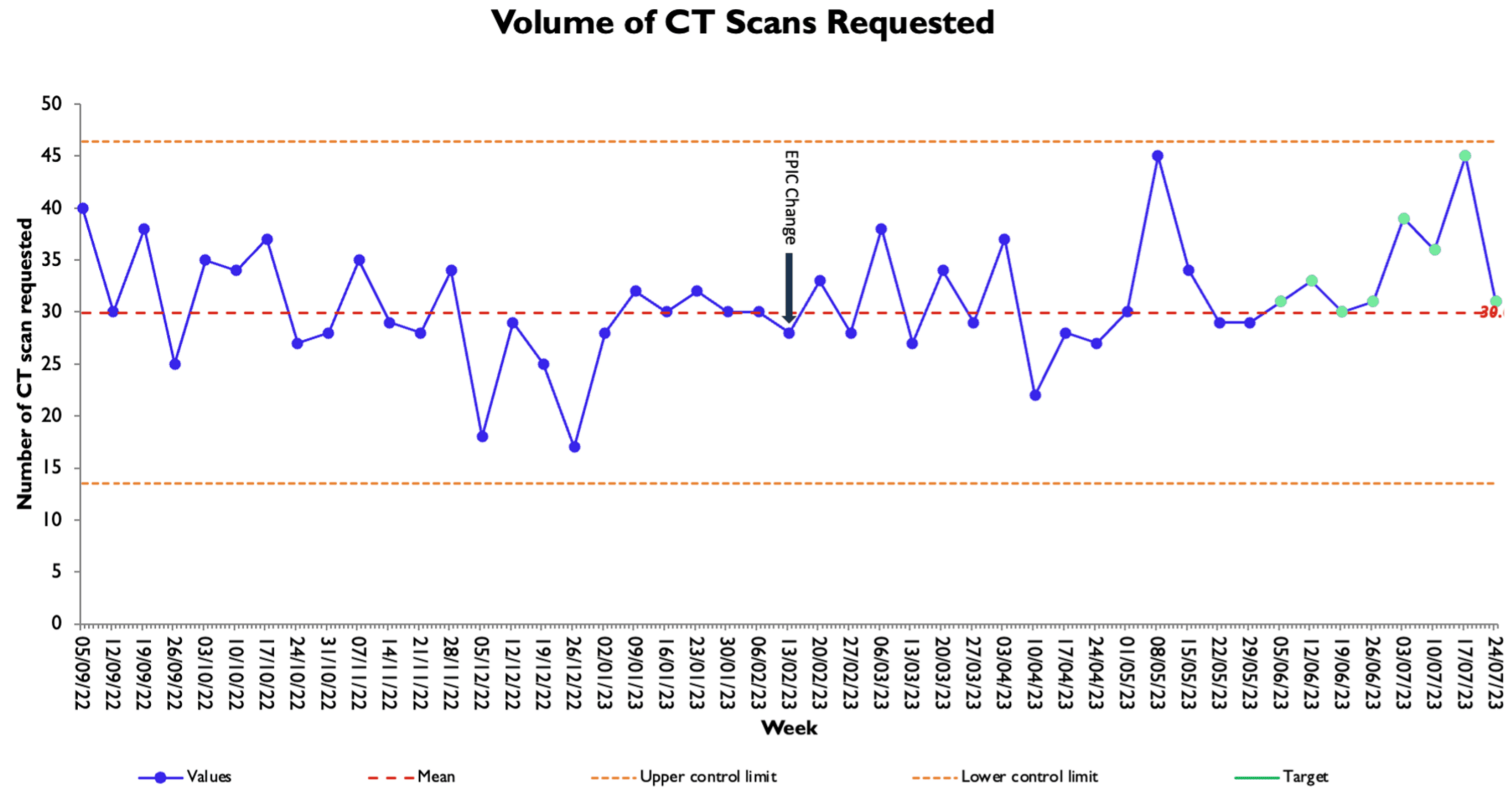
Weekly number of CT scan requests at baseline and following intervention 3 CT: computed tomography

The weekly volume of CT scans requested in the SAU is shown in Figure 3. Baseline data of CT scan requests were collected over six months prior to the implementation of intervention 3 and showed an average of 30 scans requested each week, with a range of 17-40 requests. Following the introduction of intervention 3, a further six months of data were collected. There was no significant change to the number of requests following intervention 3 (p=0.11), with an average of 32 scans per week and a range of 22-45.

## Discussion

This project aimed to reduce the inappropriate use of AXRs for acute abdominal pain in an SAU to achieve a 10% total request rate. We have demonstrated that through our three interventions, consisting of local AXR guidelines, posters, and alteration of the electronic AXR order request, inappropriate request rates have improved in our SAU. Overall, the inappropriate AXR request rate has decreased from a baseline of 69% to 11% at the end of data collection. Intervention 1 demonstrated a small improvement in inappropriate requests to 50%, further increasing to 41% after intervention 2, and following intervention 3, we see the inappropriate rate now varies between 0% and 20%. Overall numbers of monthly AXR requests reduced during the project from a monthly rate of 30 to 22. We, however, see that numbers were trending upwards towards the end of the project, which might reflect better uptake of the guidelines, given the improved concurrent appropriate request rate. There was no change in the number of CT scans required, suggesting a large volume of the previously requested AXR was being unnecessarily performed.

The majority of requests at baseline were for the suspicion of BO (60%); this reflects the previous findings in the NCEPOD and NASBO reports that AXRs were still being performed for BO [6, 7]. Following our interventions, we see a significant improvement in the rate of BO requests, improving to 5% after intervention 3. The residual BO requests might reflect the presence of BO as an option on our EPIC request form. This was recommended by radiology due to their concern over possibly increasing CT requests with the form changes. We aimed to counter this by having a further drop-down option appear when selecting BO that highlights CT imaging as the recommended investigation for diagnosing BO (Figure 1b).

Renal stone monitoring and gastrograffin follow-through studies are now the main requests, along with foreign bodies; the overall number of these has remained relatively static on a monthly basis. These requests are not typically first-line investigations for acute abdominal pain and are characteristically used as follow-up scans in small BO (SBO) for gastrograffin studies and to assess if renal stones are visible for further outpatient management. The increased number of gastrograffin studies performed might reflect an increased uptake of NASBO recommendations in managing SBO conservatively [7]. Constipation rates have remained relatively static throughout the project, from 4.4% at baseline to 4.55% after intervention 3. Previous studies assessing AXR in ED have shown that most patients diagnosed with constipation had a normal AXR and that performing imaging had no change on the patient’s management [10]. This could represent a further target for future teaching interventions to help improve the request rates. Such interventions could be through enhancing the relationship with radiographers, allowing them to reject referrals where the indication is for a constipation diagnosis only.

A number of studies have shown that inappropriate AXRs are mainly due to a lack of guideline awareness, with one study showingthat only 32% of clinicians were aware [11]. Similar studies have seen comparable results to reduce inappropriate AXR requests in ED by improving awareness of the iRefer guidelines [12,13].

Importantly, during this project, we required engagement with the trust's radiology and radiography teams as stakeholders to introduce intervention 3 and design the new AXR request form on EPIC. The primary concern from radiology was a potential increase in CT imaging requests, mostly for BO. We have, however, shown that this was not the case and that request volume remained similar before and after the intervention, though a small increase would be unlikely to be detected. This might highlight that patients prior to the interventions were having multiple scans (AXR and CT), as suggested by the national SBO audit; as such, this change will have an improvement in resource utilisation and prevent unnecessary irradiation to patients.

Despite our interventions, we still see periodic variation in our inappropriate requests. This is an important limitation and likely to be multifactorial due to locum doctors not being familiar with local policy, junior doctors rotating in the department throughout the year, or even professional disagreement with these local guidelines. A target for future interventions could remove the ‘other’ option from the X-ray request form; this may help to remove the non-specific requests, such as ‘abdominal pain’, from being requested. However, there may be specific speciality requests in which AXRs may be required, though not included in the original iRefer or our local guidelines, and can be encompassed under the other heading on the form to be scrutinised by the imaging radiographers. The duration of the project over a number of years and involvement of the hospital’s electronic system will allow the changes to be maintained within the department.

As a result of the iterative process with multiple interventions introduced, we have shown increasing improvements in the inappropriate AXR requests. We believe the key to this project was integrating our changes within the EPIC hospital system and the inclusion of the radiology department to allow the project changes implemented to be maintained in the long term. To accompany this, we now provide a teaching session on AXRs to the rotating surgical juniors to make them aware of the local policies. Although the project did not meet its target rate of 10% for inappropriate requests, it has come close to this value and has made a significant improvement from the baseline rate. This project is easily reproducible and could be implemented at other trusts to ensure that AXRs are being used appropriately and reduce the risk of harm to patients.

## Conclusions

This project has demonstrated improvement in the adherence to guidelines for the requesting of AXRs in acute abdominal pain on an SAU. This utilised three different interventions to enable this change. AXR requests for BO reduced significantly with no increase in concurrent CT scans, whereas overall AXR request volume decreased. Though it has not achieved its target reduction, given the span of time this project was conducted over, we believe this change will be sustained within the trust with the inclusion of the radiology department and utilisation of the electronic hospital system. These changes will reduce excessive patient irradiation and unnecessary waste of hospital resources.
